# ICG-001 suppresses growth of gastric cancer cells and reduces chemoresistance of cancer stem cell-like population

**DOI:** 10.1186/s13046-017-0595-0

**Published:** 2017-09-11

**Authors:** Yi Liu, Hui Chen, Peiming Zheng, Yingxia Zheng, Qin Luo, Guohua Xie, Yanhui Ma, Lisong Shen

**Affiliations:** Department of Clinical Laboratory, Xinhua Hospital, Shanghai Jiao Tong University School of Medicine, Shanghai, 200092 China

**Keywords:** ICG-001, Gastric cancer, Wnt/β-catenin signaling pathway, Growth, Stem cell-like

## Abstract

**Background:**

ICG-001, a small molecule, binds CREB-binding protein (CBP) to disrupt its interaction with β-catenin and inhibits CBP function as a co-activator of Wnt/β-catenin-mediated transcription. Given its ability to inhibit Wnt/β-catenin signaling pathway, ICG-001 has been used in some tumor types to exert its anticarcinogenic effect. Here, we examined ICG-001 and its potential role as a therapeutic in gastric cancer (GC).

**Methods:**

The gastric cancer cell lines SGC-7901, MGC-803, BGC-823 and MKN-45 were used in vitro and in vivo. The abilities of cell proliferation, tumor sphere formation, metastasis, tumorgenesis and chemoresistance to chemotherapy drugs in vitro were evaluated by MTT assay, colony formation assay, flow cytometry, migration and invasion assay, and tumor spheres culture. The in vivo experiments were performed using a subcutaneous transplantation tumor model in athymic nude mice. Alterations at RNA and protein levels were followed by qRT-PCR, western blot, coimmunoprecipitations and immunofluorescence assay.

**Results:**

In this study, we showed that ICG-001 significantly inhibited growth and metastasis of multiple GC cell lines, induced cell apoptosis, and augmented in vitro tumor spheres suppression when used in combination with chemotherapy drugs probably through robustly blocking association of β-catenin with CBP and N-cadherin, but promoting association of β-catenin with P300 and E-cadherin, instead of altering the distribution and expression of β-catenin.

**Conclusions:**

Our findings suggest that ICG-001 suppresses GC cell line growth, metastasis and reduces its stem cell-like properties and chemoresistance, indicating that ICG-001 is a potentially useful small molecule therapeutic for GC.

## Background

Gastric cancer (GC) is currently the fourth most common malignancy and the third leading cause of cancer-related deaths worldwide [1]. The incidence and mortality of gastric cancer are the highest in East Asia (particularly in Korea, Mongolia, Japan, and China), and it has become the second most lethal cancer in China [2]. GC is difficult to treat because it frequently presents at an advanced, non-operative stage and is highly resistant to cytotoxic or targeted molecular therapy. While our understanding of the molecular and cellular basis of GC continues to expand, present therapeutic options remain limited and offer only modest survival benefits for most patients.

Wnt/β-catenin signaling pathway is a critical developmental signaling pathway whose deregulation is strongly implicated in the pathogenesis of many types of cancer [3]. Perturbations of Wnt/β-catenin signaling pathway can promote the initiation and progression of GC and has been linked to aggressive tumor behavior [4]. Although plagued by poor pharmacokinetics in vivo, several novel Wnt/β-catenin inhibitors have been demonstrable in vivo activity and are now in various stages of preclinical or early clinical development. ICG-001 was first identified in a screen of small molecules that inhibited Wnt/β-catenin transcriptional activity in a colorectal cancer cell line [5]. ICG-001 selectively blocks the interaction of β-catenin with its transcriptional co-activator cyclic-AMP-response-element-binding protein (CBP). Recent studies have provided convincing evidence of the inhibitory effects of ICG-001 on Wnt-driven disease models including pulmonary fibrosis [6], renal interstitial fibrosis [7], acute lymphoblastic leukemia [8], chronic myocardial infarction [9], dermal fibrosis [10], salivary tumorigenesis [11] and pancreatic ductal adenocarcinoma [12]. However, ICG-001 has not been explored in gastric cancer. Further, the mechanisms of ICG-001 in cancer inhibition and in chemoresistance of cancer stem cells to chemotherapy drugs are not yet fully discovered.

Given the importance of Wnt/β-catenin signaling pathway in GC, we have now explored therapeutic potential and related mechanism of ICG-001 in GC cell lines and stem-like cells. ICG-001 significantly inhibited in vitro and in vivo GC cell lines growth by inducing G0/G1 cell cycle arrest and reduced chemoresistance of stem-like cells to chemotherapy drugs. Mechanically, ICG-001 disrupted the association between β-catenin with CBP, P300, E-cadherin and N-cadherin, instead of perturbing the expression and distribution of β-catenin.

## Methods

### Cell culture and treatment

The gastric cancer cell lines SGC-7901, MGC-803, BGC-823 and MKN-45 were purchased from the Chinese Academy of Sciences Cell Bank of Type Culture Collection. Cells were maintained in DMEM containing 10% FBS supplemented with 100 U/mL penicillin and 100 μg/ml streptomycin (Gibco). For tumor spheres culture, cells were seeded in dishes pre-coated with 18 mg/ml polyHEMA and cultured in serum-free DMEM/F12 media supplemented with 20 ng/ml EGF, 10 ng/ml bFGF, 1% N2 and 2% B27. ICG-001 was purchased from MedChemExpress and diluted in Dimethyl Sulfoxide (DMSO).

### Cell viability and adhesion-dependent colony formation assay

Gastric cancer cells were seeded in 96-well plate at 1500–3000 cells per well and incubated with ICG-001 for 0–6 days, and cell viability was detected with 3-(4,5-dimethyl-2-thiazolyl)-2,5-diphenyl-2-H-tetrazolium bromide (MTT) (Sigma-Aldrich). The optical density at 490 nm was measured on a multiwall plate reader (FLX800, Bio-TEK). Gastric cancer cells were plated in 60-mm dishes at a density of 2 × 10^3^ cells per well for adhesion-dependent colony formation assay. ICG-001 was added to the culture medium at different concentrations diluted with DMSO, and based on MTT results, the final concentraton was 25 μM. Culture medium was changed every 3–4 days. Then, 3–4 weeks later, the remaining colonies were fixed with 4% paraformaldehyde and dyed with crystal violet. The colonies were counted according to the defined colony size.

### Flow cytometry

GC cell lines and tumor spheres treated with ICG-001 for 48 h were trypsinized and washed twice in 1 × PBS, after being resuspended in 100 μl FBS, fluorochrome-conjugated antibodies against CD44 and their respective isotype controls were added to stain for 30 min at 4 °C. Following being washed twice in 1 × PBS, labeled cells were analyzed by flow cytometry on a FACS Canto II flow cytometer (BD Biosciences) and the results were analyzed with FlowJo software (Tree Star).

### Cell cycle analysis

Cell cycle was analyzed using BD Cycletest Plus DNA Reagent Kit (BD Pharmagen, USA) following the manufacturer’s protocol [13].

### Apoptosis assay

Apoptosis was measured using FITC Annexin V Apoptosis Detection Kit I (BD Pharmagen, USA) following the manufacturer’s protocol. In brief, cells were washed twice with cold PBS and then resuspended in 100 μl of 1× Binding Buffer, then 5 μl of FITC Annexin V and 5 μl of propidium iodide (PI) were added to stain for 15 min at room temperature in the dark. After incubation 400 μl of 1× Binding Buffer were added to each tube and the cells were analyzed by FACS Canto II flow cytometry (BD Biosciences).

### RNA extraction and quantitative real-time PCR

Total RNA was extracted using TRIzol reagent (Invitrogen, USA) according to the manufacturer’s instructions. The concentration and quality of the total RNA were assessed with Nanodrop Spectrophotometer (Thermo Fisher Scientific, USA). For the mRNA expression analysis, reverse transcription was performed using PrimeScript RT master mix (TaKaRa, Japan). Quantitative real-time PCR analysis was performed in triplicate on 7900 HT Real-Time PCR System (Applied Biosystems, USA) using SYBR Premix Ex Taq (TaKaRa, Japan) and the expression level of ACTIN was used as endogenous control. Results were analyzed using the 2^–ΔΔct^ calculation method.

### In vitro cell migration and invasion assay

Cell migration and invasion assays were conducted on 24-well Transwell cell chambers with 8-μm sized pores as previously described [13].

### Cell nuclear and cytoplasmic extraction

Cell nuclear and cytoplasmic proteins were extracted using the nuclear and cytoplasmic extraction reagents (Thermo, USA). Cells were added CER I: CER II: NER reagents at 200: 11: 100 μL, then the nuclear and cytoplasmic proteins were extracted respectively.

### Western blot

The cells were lysed in equal volumes of ice cold lysis buffer with protease inhibitor cocktail. Cell lyses were separated by SDS-PAGE and then transferred to a 0.2-μm PVDF membrane (Bio-Rad, USA). After being blocked with Odyssey Blocking Buffer (Li-COR Biosciences, USA), the membrane was incubated with primary antibody (1:1000) at 4 °C overnight, followed by incubation with IRDye 800CW or 680 secondary antibodies (1:5000, LI-COR Biosciences, USA). ACTIN was used as endogenous control. The Odyssey Infrared Imaging System was used to visualize targeted protein bands.

### Coimmunoprecipitations

For β-catenin-CBP/P300/E-cadherin/N-cadherin coimmunoprecipitations, MGC-803 cells were incubated 48 h with or without ICG-001 and 100 μg protein extract was diluted to 1 mL in coimmunoprecipitation (Co-IP) buffer (Thermo, USA). 2 μg of β-catenin (Santa Cruz Biotechnology, USA) antibody was added to the protein samples and the mix were incubated overnight at 4 °C with rotation. 20 μL of 50% protein A/G-agarose bead slurry (equilibrated in Co-IP buffer) were added, and after 2 h–incubation at 4 °C, the beads were washed 4 times with Co-IP buffer (1 ml per wash) and diluted with 1× loading buffer. Western blotting was performed, and CBP (Santa Cruz Biotechnology, USA), P300 (Santa Cruz Biotechnology, USA), E-cadherin (Cell Signaling Technology, USA), N-cadherin (Cell Signaling Technology, USA) were detected.

### Immunofluorescence assay

Specimens were prepared as previously described [14]. Images were captured using a Leica SP5 Laser scanning confocal microscope.

### In vivo xenograft and treatment experiments

For in vivo studies, 4–6 week old male nude mice were purchased from Shanghai Laboratory Animal Center of China. MGC-803 cells (2 × 10^6^ cells in 200 μl PBS) were subcutaneously injected into right flank of the nude mice to establish tumors. After 7 days, 50 mg/kg/day ICG-001 was delivered into the tumors. PBS was used as the control. Treatment continued for a total of 4 weeks. The tumor size was measured using digital caliper and tumor volume was calculated with the following formula: volume = 0.5 × width2 × length. All animal procedures were carried out with the approval of the Institutional Committee of Shanghai Jiao Tong University School of Medicine for Animal Research.

### Statistical analysis

Statistical significance between groups was determined by two-tailed Student’s t-test and one-way ANOVA test. Differences were considered to be significant when *P* < 0.05. All statistical data were displayed as means ± standard deviation (SD) and analyzed for statistical significance with GraphPad Prism 5.0 for Windows (GraphPad Software, USA).

## Results

### ICG-001 inhibits in vitro gastric cancer cell line growth

**Fig. 1:**
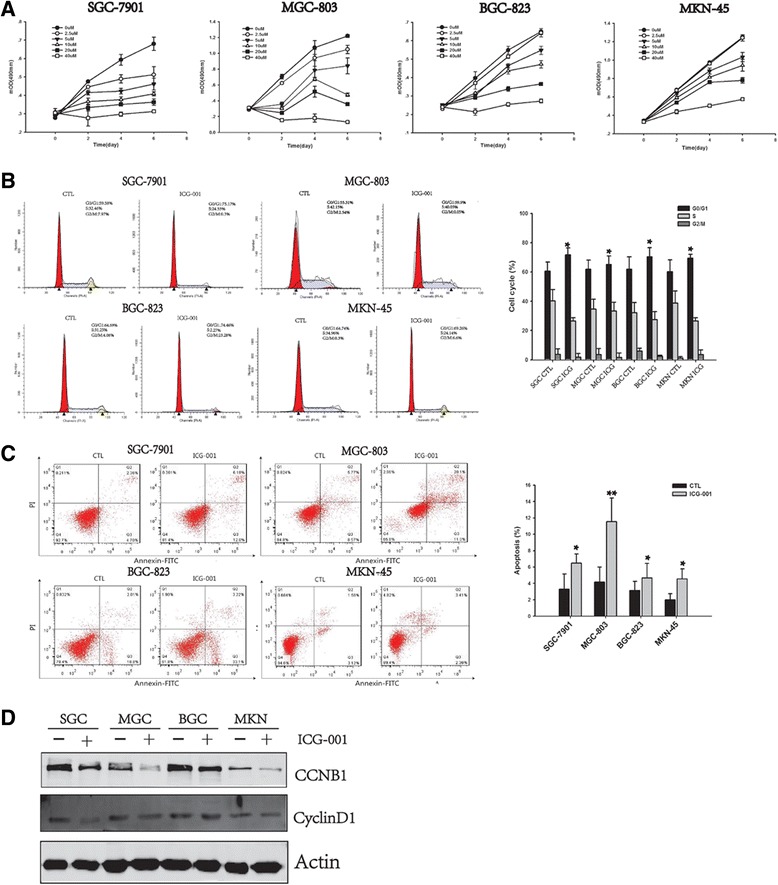
ICG-001 inhibits in vitro gastric cancer cell line growth. **a** MTT cell viability was performed at different concentrations of ICG-001. **b** Cell cycle and **c** apoptosis were analyzed by flow cytometry. **d** ICG-001 reduced expression of CCNB1 and CyclinD1 in MGC-803. Data are the mean ± SEM of three independent experiments. * *p* < 0.05, ** *p* < 0.01, *** *p* < 0.001 vs. control

ICG-001 effects on anchorage-dependent gastric cancer cell growth in vitro were addressed by MTT assay. ICG-001 showed dose- and time-dependent cell growth inhibition of SGC-7901, MGC-803, BGC-823 and MKN-45 cell lines at concentration of 26.6 μM, 8.8 μM, 26.4 μM and 31.8 μM, respectively (Fig. 1a). To explore mechanisms underlying ICG-001 gastric growth inhibitory effects, we next performed cell cycle and apoptosis analysis on SGC-7901, MGC-803, BGC-823 and MKN-45 cell lines after 48 h’ treatment with ICG-001. As Fig. 1b and c showed that ICG-001 increased the percentage of G0/G1 phrase and apoptosis cells. Further, ICG-001 treatment decreased the expression cell cycle proteins such as CCNB1 and CyclinD1 in these GC cell lines (Fig. 1d).

### ICG-001 inhibits gastric cancer stem-like cells self-renewal properties

**Fig. 2:**
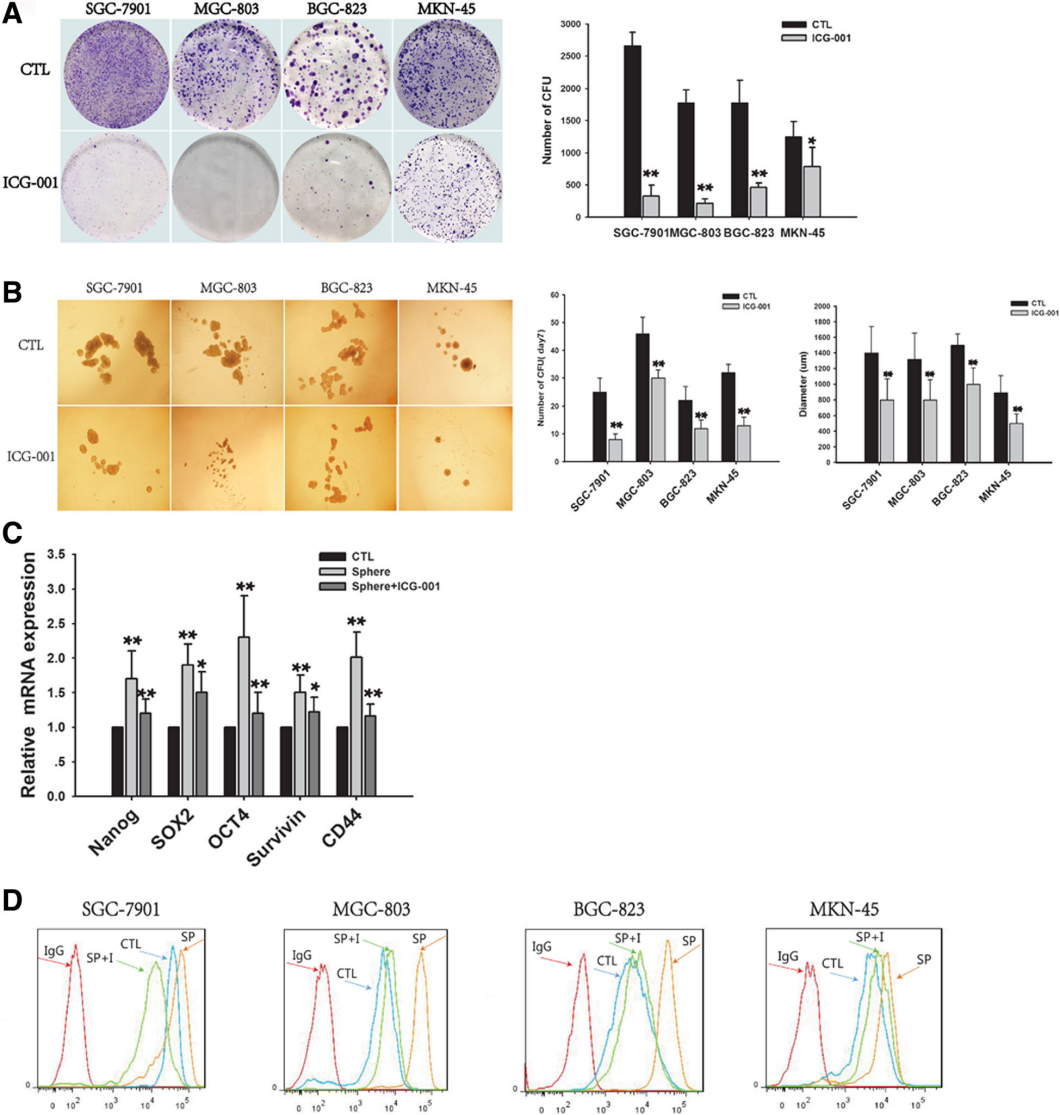
ICG-001 inhibits gastric cancer stem-like cells self-renewal properties. **a** ICG-001 inhibited the colony formation capacity and **b** tumor sphere formation capacity in four cell lines. Representative photograph of colony and tumor spheres were shown on the left. Statistical analysis was shown on the right. Several gastric stem cell markers expression were measured by **c** qRT-PCR and **d** flow cytometry. Data are the mean ± SEM of three independent experiments. * *p* < 0.05, ** *p* < 0.01, *** *p* < 0.001 vs. control

We also assayed the colony formation capacity in above cell lines under the treatment of ICG-001. The results showed 25 μM ICG-001 significantly inhibited the colony formation capacity in these cell lines as shown in Fig. 2a. The tumor sphere formation assay was employed to study the inhibitory effect of ICG-001 on the growth of gastric cancer stem-like cells. In this study, SGC-7901, MGC-803, BGC-823 and MKN-45 cell lines were incubated with 25 μM ICG-001 for 7 days and the number and diameter of tumor spheres were quantified. Results in Fig. 2b showed that ICG-001 greatly suppressed the formation of tumor spheres. A substantial inhibition of both size and number of tumor spheres were displayed after the treatment of ICG-001. Moreover, we also compared gastric cancer stem cells marker expression, such as OCT-4, Sox2, Nanog, Survivin and CD44, in tumor spheres and tumor spheres treated with ICG-001, which showed those markers were higher in tumor spheres than cell lines, but declined after ICG-001 treatment (Fig. 2c and d).

### ICG-001 inhibits in vivo gastric tumor growth

**Fig. 3:**
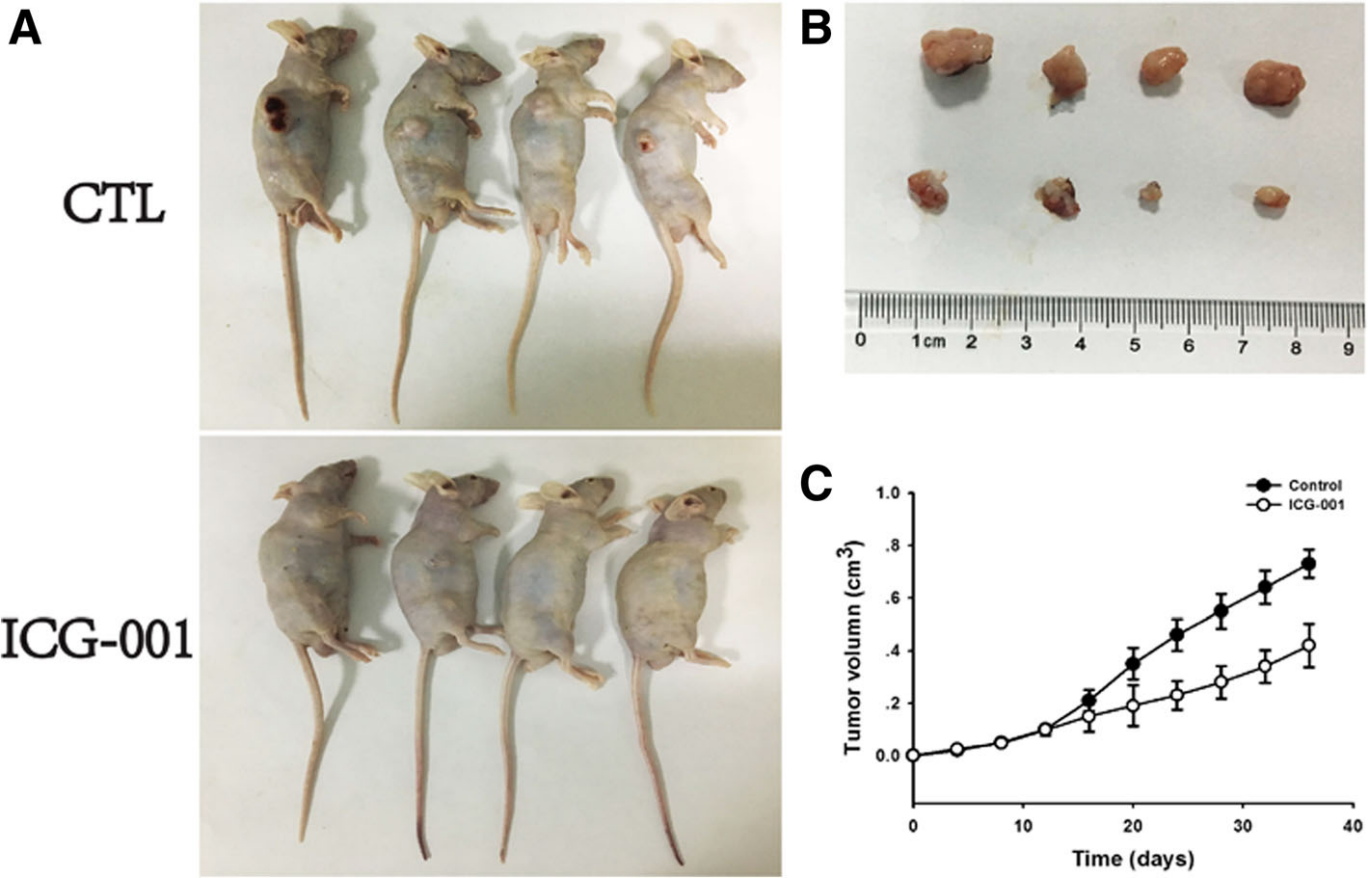
ICG-001 inhibits in vivo gastric tumor growth. **a** and **b** Tumors were produced by MGC-803. MGC-803 Cell (2× 10^6^) were injected subcutaneously in the right flank of nude mice per mouse respectively (*n* = 4). And when the tumors developed in 7 days, the mice were randomly distributed into two groups, and were untreated or treated with ICG-001 (50 mg/kg/day); **c** Tumor growth curves were monitored during the experimental period (*n* = 40). Data represent the mean ± SEM of three independent experiments. * *p* < 0.05, ** *p* < 0.01, *** *p* < 0.001 vs. control

ICG-001 was next addressed in the MGC-803 xenograft models. MGC-803 cells were subcutaneously inoculated into the right flanks of nude mice. When the tumors became palpable, 50 mg/kg/day ICG-001 was delivered into the tumors. PBS was used as the control. Treatment was continued for a total of 4 weeks, after which the tumor volumes were measured and plotted as the average (Fig. 3). As the results showed that the smaller tumor volumes were observed in ICG-001 treatment group. ICG-001 significantly reduced tumor volumes compared to the control group by xenograft models analysis.

### ICG-001 prevents gastric cancer cell lines migration and invasion

**Fig. 4:**
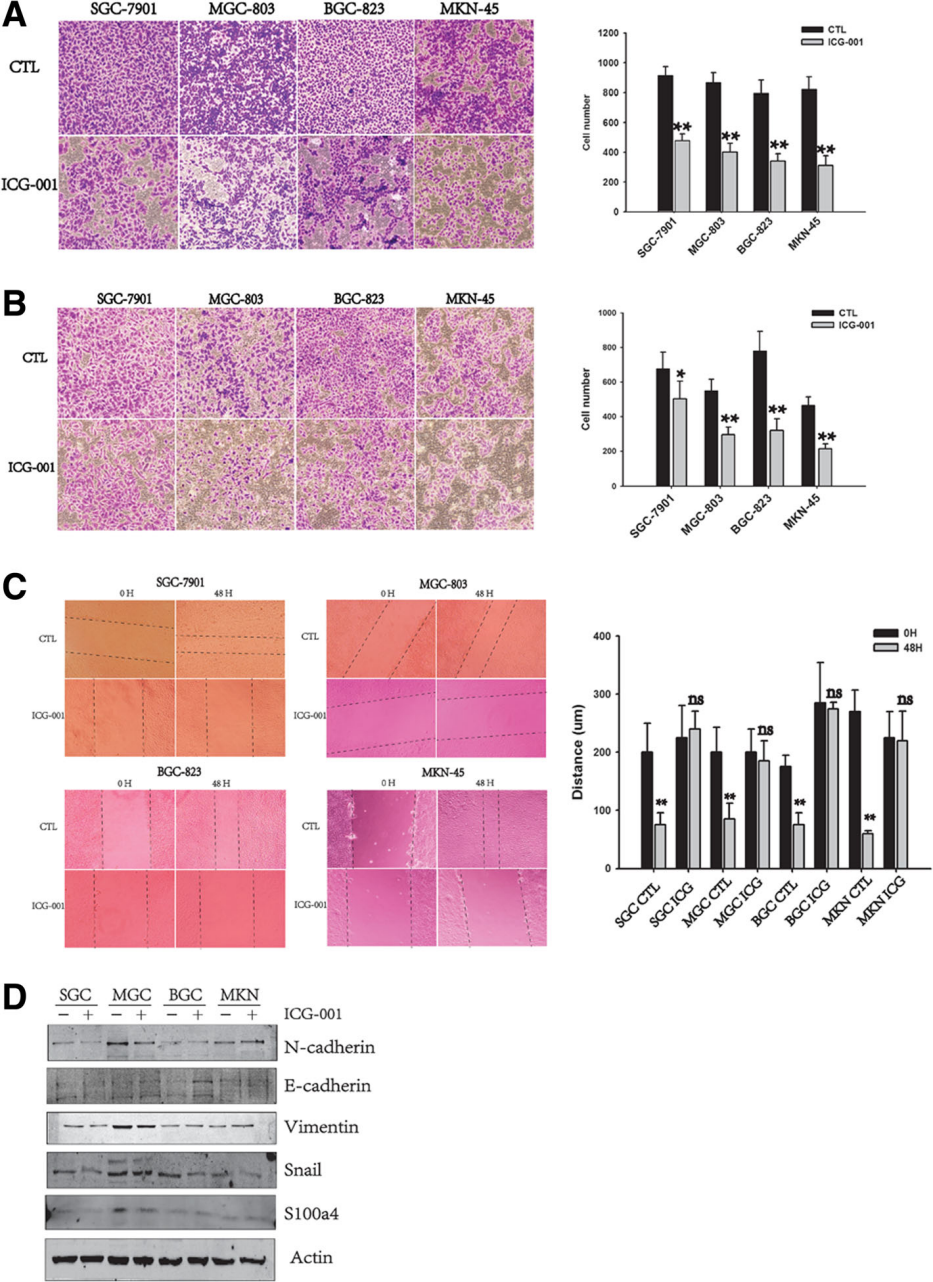
ICG-001 prevents gastric cancer cell lines migration and invasion. **a** and **b** ICG-001 significantly inhibited GC cells migration and invasion. Representative photograph of migrated cells were shown on the left. Statistical analysis was shown on the right. **c** Analysis of wound repair at 0 h and 48 h after scratch wounding, using ImageJ software. **d** Western blotting analysis of epithelial protein and mesenchymal proteins expression after ICG-001 treatment for 48 h. Data represent the mean ± SEM of three independent experiments. * *p* < 0.05, ** *p* < 0.01, *** *p* < 0.001 vs. control

ICG-001 not only has been shown to inhibit cell growth, but also prevents EMT (epithelial mesenchymal transition). To determine the effects of ICG-001 on cell migration and invasion, we treated cells with ICG-001 for 48 h, and the migrated cells through inserts were counted. The wound healing assay was also used to analyze the effects of ICG-001 on migration. Exposure to ICG-001 significantly depletes the migration and invasion ability of gastric cancer cells (Fig. 4a–c). We then sought to explore the potential mechanisms underlying the inhibitory effect of ICG-001 on cell migration and invasion. As Fig. 4d showed that the expression of E-cadherin and N-cadherin had no significant changes in most cells after ICG-001 treatment, as well as other proteins, such as vimentin, sail and a member of S100 calcium-binding protein family secreted by tumor (S100A4). Therefore, the potential mechanisms underlying the inhibitory effect of ICG-001 on cell migration and invasion needed to be explored further.

### ICG-001 reduces gastric cancer stem-like cells chemoresistance

**Fig. 5:**
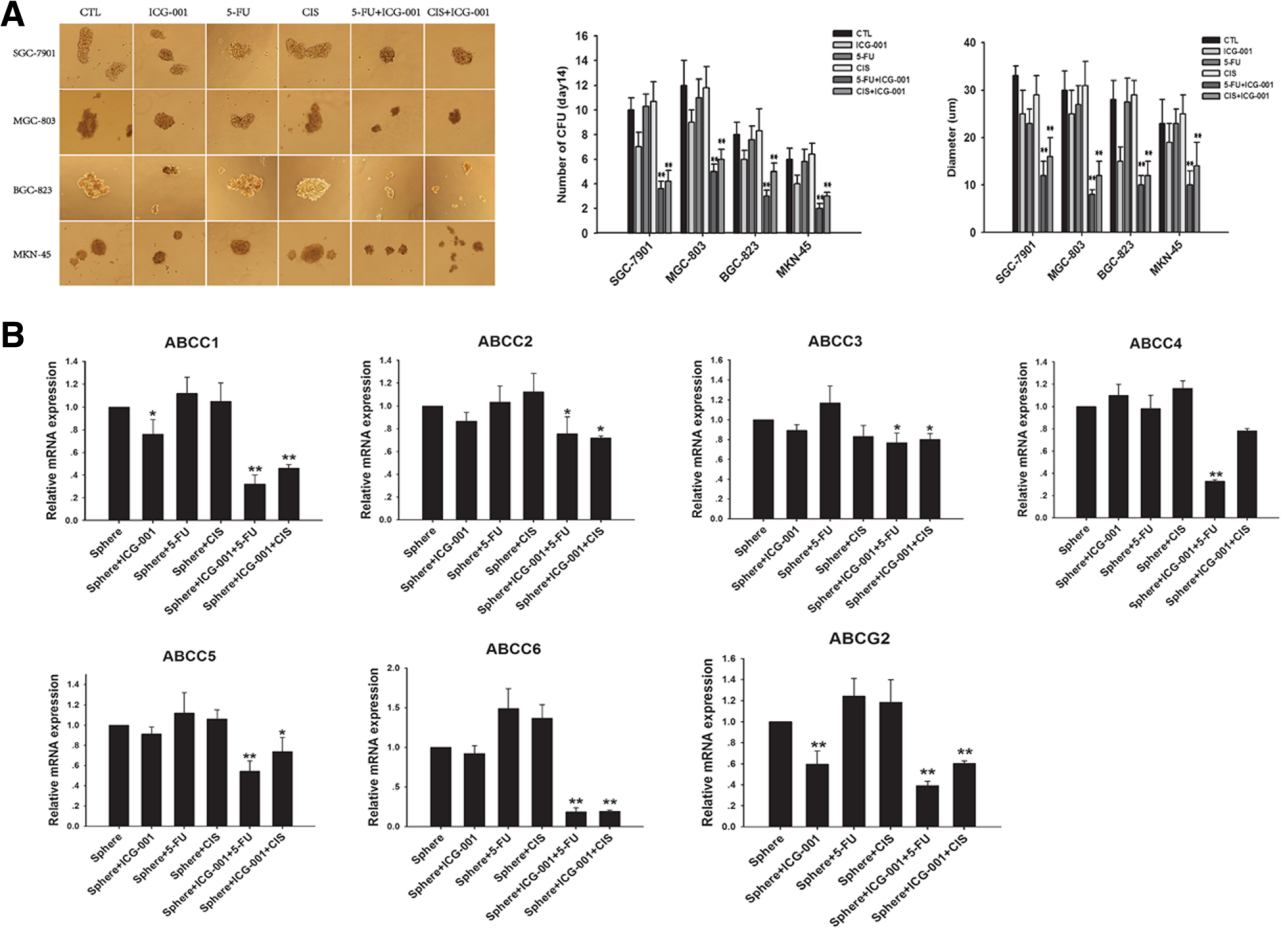
ICG-001 reduces gastric cancer stem-like cells chemoresistance. **a** Effect of ICG-001 on the growth of established spheroids. Tumor spheres were allowed to form for 7 days, and then the established tumor spheres were incubated with ICG-001 and 5-Fu or cisplatin for another 7 days. The number and size of tumor sphere formed from GC cell lines with and without ICG-001 treatment were compared. **b** Drug resistant genes expression in MGC-803 under the treatment of ICG-001(25 μM). Data represent the mean ± SEM of three independent experiments. * *p* < 0.05, ** *p* < 0.01, *** *p* < 0.001 vs. control

Chemoresistance is an important characteristic of cancer stem cells. Then we next investigated whether ICG-001 would reduce the chemoresistance of gastric cancer stem-like cells. For this purpose, 5-Fu and cisplatin were used to treat cells. As shown in Fig. 5a, ICG-001 could significantly enhance the chemosensitivity of cancer stem-like cells to chemotherapy drugs. Moreover, we detected the chemoresistant genes, such as ABCC1, ABCC2, ABCC3, ABCC4, ABCC5, ABCC6 and ABCG2. All these genes were reduced after the treatment of ICG-001 in combination with 5-Fu or cisplatin (Fig. 5b).

### ICG-001 mediates the association of β-catenin with CBP, P300, E-cadherin and N-cadherin

**Fig. 6:**
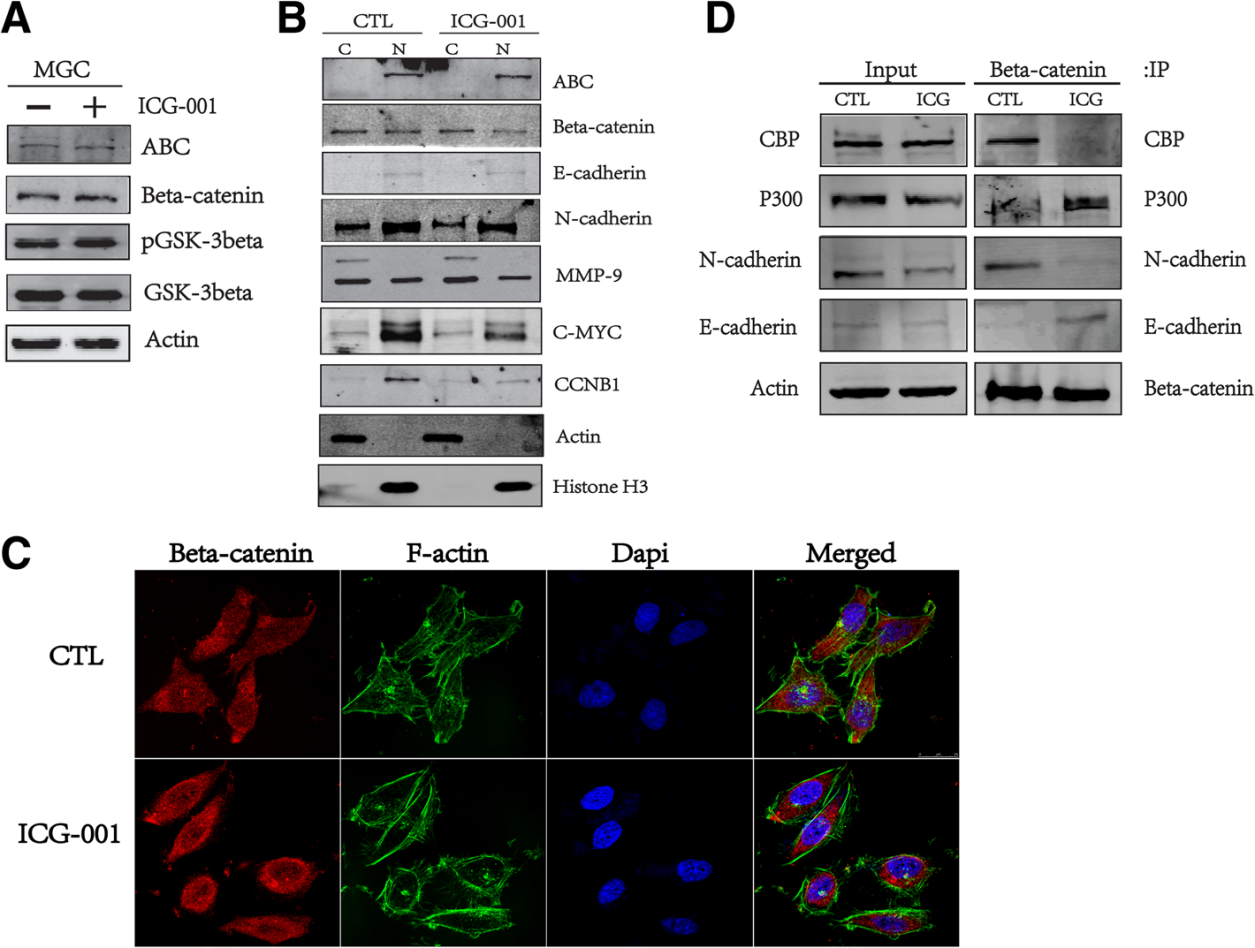
ICG-001 mediates the association of β-catenin with CBP, P300, E-cadherin and N-cadherin. **a** and **b** Western blotting analysis of Wnt related proteins from total proteins and extracted nuclear and cytoplasmic proteins. **c** Confocal analysis of the expression and distribution of β-catenin in MGC-803 with or without ICG-001. **d** Coimmunoprecipitation of MGC-803 cells, performed as described in Materials and Methods, using 25 μM ICG-001. Anti-β-catenin, anti-CBP, anti-P300, anti-N-cadherin or anti-E-cadherin antibodies were used for the immunoprecipitation

To further explore the mechanisms of ICG-001 effects on cell growth, we tested Wnt/β-catenin signaling pathway activity and the association of β-catenin with CBP, P300, E-cadherin and N-cadherin. As we expected, ICG-001 treatment did not affect the expression of β-catenin and GSK-3β, as well as β-catenin distribution. However, a set of proteins related with cell proliferation and EMT were regulated by ICG-001, including N-cadherin, MMP-9, C-MYC, and CCNB1 (Fig. 6a and b). After treatment with ICG-001, the cell morphology turned round with fewer tentacles (Fig. 6c). In addition, we found that ICG-001 influenced Wnt/β-catenin signaling pathway activity through mediating the associations of β-catenin with CBP, P300, E-cadherin and N-cadherin. As Fig. 6d showed, ICG-001 treatment attenuated the associations of β-catenin/CBP and β-catenin/N-cadherin, but enhanced the associations of β-catenin/P300 and β-catenin/E-cadherin.

## Discussion

Wnt/β-catenin signaling pathway is a pivotal signaling pathway regulating cancer cell growth and cell migration and invasion [15, 16]. GC cells are dependent upon Wnt/β-catenin signaling pathway for initiation and progression, warranting exploration of ICG-001 and Wnt other inhibitors as potential therapeutics [17, 18]. We have demonstrated that ICG-001 significantly inhibits in vitro GC cell line growth through induction of G0/G1 cell cycle arrest and apoptosis and reduction of tumor volume in an in vivo xenograft model of GC. Furthermore, ICG-001 was shown to inhibit gastric cancer stem-like cells self-renewal properties. On the other hand, a variety of studies have suggested that cancer cell migration and invasion are regulated by Wnt/β-catenin signaling pathway. In our present study, we showed that ICG-001 prevented gastric cancer cell lines migration and invasion and the potential mechanisms underlying the inhibitory effect of ICG-001 on cell migration and invasion were related with the variation of epithelial protein and mesenchymal protein levels. These results supported our hypothesis that ICG-001, as an inhibitor of Wnt/β-catenin signaling pathway, could exert the antitumor effects in GC. Thus, ICG-001 may offer survival and therapy benefit in GC or other tumors.

Cancer stem cells (CSCs) represent a subpopulation of cells within the bulk tumor that have capability to undergo self-renewal, drive the tumor growth, and are resistant to radiation and conventional drugs [19–21]. The residual CSCs after conventional treatment can lead to tumor relapse in local and distant regions. Therapeutic targeting of the CSC population may overcome the relapse and distant metastatic treatment failure in GC. The existence of CSCs has recently been identified in various types of solid tumors such brain tumor, breast cancer, ovarian cancer, and GC [22–26]. In GC, CD44 expression was found to be enriched in the CSC population and serves as a potential marker for the CSC population [27, 28]. In our study, we demonstrated that ICG-001 could inhibit the growth of tumor spheres and reduce the expression of CD44 as well as other stem cell markers such as OCT-4, Sox2, Nanog, Survivin. Chemoresistance is an important characteristic of CSCs [29, 30]. Compared with control group, tumor spheres were relatively resistant to 5-Fu and cisplatin. Our results showed that ICG-001 enhanced gastric cancer stem-like cells chemosensitivity to 5-Fu and cisplatin. The chemosensitivity upregulation is associated with downregulation of the ABC (ATP binding cassette) family of membrane transport proteins, comprising of seven subfamilies ranging from A to G. These proteins play an important role in cancer chemoresistance. Certain ABC transporters couple the hydrolysis of ATP to move drug and xenobiotics unidirectionally out of cells, thereby effecting drug resistance [31]. These results strongly suggest that combination of ICG-001 with chemotherapy drugs can enhance the tumor suppressive effect in GC cells.

Mechanistically, we showed that ICG-001 inhibited Wnt/β-catenin signaling pathway activity without changing the expression and distribution of β-catenin, but affecting its downstream target proteins and the associations between β-catenin with CBP, P300, N-cadherin and E-cadherin. The differential effects of Wnt/β-catenin signalling on target genes may be determined by different co-activators. By targeting the critical point of nuclear β-catenin with its transcriptional co-activator CBP, ICG-001 affects the cell fate of maintaining pluripotency or initiating differentiation [32, 33]. ICG-001 inhibits Wnt transcriptional activity by binding to the N-terminal domain of CBP and preventing its interaction with β-catenin [34]. This not only has the potential to disrupt CBP-dependent β-catenin-TCF transcription, but also may increase the pool of β-catenin available to interact with P300. Like CBP, P300 functions either as a transcriptional co-activator or co-repressor in a context-dependent manner [5]. In the present study, we found that ICG-001 attenuated the association of β-catenin with CBP, but augmented the association of β-catenin with P300. Thus, transcription driven by β-catenin/P300 interaction initiated a program that promoted differentiation, which could explain the effects of ICG-001 on cancer stem-like cells, enhanced chemosensitivity to chemotherapy drugs. N-cadherin and E-cadherin regulate the epithelial integrity and tissue architecture by maintaining cell-cell junctions between adjacent cells. During EMT epithelial-type cancer cells undergo a set of molecular, morphological and functional changes with loss of E-cadherin and gain of N-cadherin, leading to impaired epithelial cell-cell junctions and cell polarity, acquisition of a mesenchymal motile cell phenotype. These changes facilitate cancer cells migration, intravasation and dissemination. Studies in cancer cells support the pivotal role of N-cadherin and E-cadherin in EMT [35]. Data generated by our group showed that ICG-001 effectively abolished the N-cadherin/β-catenin association and promoted E-cadherin/β-catenin association, thereby inhibiting morphological changes of EMT, cell migration and invasion.

## Conclusions

In summary, ICG-001 appears to robustly inhibit GC growth and to reduce cancer stem-like cells chemoresistance through both direct and indirect mechanisms. While further studies are needed to expand upon precise mechanisms underpinning its transcriptional and phenotypic activities, ICG-001 appears to more broadly disrupt CBP function beyond its effects as co-transcriptional activator of Wnt/β-catenin signaling. Future studies should not only further explore the therapeutic potential of ICG-001 and its derivatives in GC, but also better establish effective drug combinations and patient selection.

## Abbreviations

ABC: Active β-catenin
ABC: ATP binding cassette
CBP: Cyclic-AMP-response-element-binding protein
Co-IP: Coimmunoprecipitation
CSCs: Cancer stem cells
EMT: Epithelial mesenchymal transition
FBS: Fetal bovine serum
GC: Gastric cancer
MTT: 3-(4,5-dimethyl-2-thiazolyl)-2,5-diphenyl-2-H-tetrazolium bromide
PBS: Phosphate buffer saline
qRT-PCR: Quantitative Real-time PCR

## References

[1] Torre LA, Bray F, Siegel RL, Ferlay J, Lortet-Tieulent J, Jemal A (2015). Global cancer statistics, 2012. CA Cancer J Clin.

[2] Chen W, Zheng R, Baade PD, Zhang S, Zeng H, Bray F, Jemal A, Yu XQ, He J (2016). Cancer statistics in China, 2015. CA Cancer J Clin.

[3] Clevers H (2006). Wnt/beta-catenin signaling in development and disease. Cell.

[4] White BD, Chien AJ, Dawson DW (2012). Dysregulation of Wnt/β-catenin signaling in gastrointestinal cancers. Gastroenterology.

[5] Emami KH, Nguyen C, Ma H, Kim DH, Jeong KW, Eguchi M (2004). A small molecule inhibitor of β-catenin /CREB-binding protein transcription. Proc Natl Acad Sci U S A.

[6] Henderson WR, Chi EY, Ye X, Nguyen C, Tien Y, Zhou B (2010). Inhibition of Wnt/beta-catenin/ CREB binding protein (CBP) signaling reverses pulmonary fibrosis. Proc Natl Acad Sci U S A.

[7] Hao S, He W, Li Y, Ding H, Hou Y, Nie J (2011). Targeted inhibition of β-catenin/CBP signaling ameliorates renal interstitial fibrosis. J Am Soc Nephrol.

[8] Gang EJ, Hsieh Y-T, Pham J, Zhao Y, Nguyen C, Huantes S (2014). Small-molecule inhibition of CBP/catenin interactions eliminates drug-resistant clones in acute lymphoblastic leukemia. Oncogene.

[9] Sasaki T, Hwang H, Nguyen C, Kloner R a, Kahn M (2013). The small molecule Wnt signaling modulator ICG-001 improves contractile function in chronically Infarcted rat myocardium. PLoS One.

[10] Beyer C, Reichert H, Akan H, Mallano T, Schramm A, Dees C (2013). Blockade of canonical Wnt signalling ameliorates experimental dermal fibrosis. Ann Rheum Dis.

[11] Wend P, Fang L, Zhu Q, Schipper JH, Loddenkemper C, Kosel F (2013). Wnt/β-catenin signaling induces MLL to create epigenetic changes in salivary gland tumours. EMBO J.

[12] Arensman MD, Telesca D, Lay AR (2014). The CREB binding protein inhibitor ICG-001 suppresses pancreatic cancer growth. Mol Cancer Ther.

[13] Chen H, Xie GH, Wang WW, Yuan XL, Xing WM, Liu HJ (2015). Epigenetically downregulated Semaphorin 3E contributes to gastric cancer. Oncotarget.

[14] Yuan X, Yu L, Li J, Xie G, Rong T, Zhang L (2013). ATF3 Suppresses metastasis of bladder cancer by regulating gelsolin-mediated remodeling of the actin cytoskeleton. Cancer Res.

[15] Yunlong Ma, Bin Zhu, Xiaoguang Liu, et al. Inhibition of oleandrin on the proliferation show and invasion of osteosarcoma cells in vitro by suppressing Wnt/β-catenin signaling pathway. J Exp Clin Cancer Res. 2015;34:115. 10.1186/s13046-015-0232-8 PMC4596494 26444270

[16] Takahashi-Yanaga F, Kahn M (2010). Targeting Wnt signaling: can we safely eradicate cancer stem cells?. Clin Cancer Res.

[17] Abdel-Magid AF (2014). Wnt/β-catenin signaling pathway inhibitors: a promising cancer therapy. ACS Med Chem Lett.

[18] Yao H, Ashihara E, Maekawa T (2011). Targeting the Wnt/β-catenin signaling pathway in human cancers. Expert Opin Ther Targets.

[19] Shuka G, Khera HK, Srivastava AK (2017). Therapeutic potential, challenges and future perspective of cancer stem cells in translational oncology: a critical review. Curr Stem Cell Res Ther.

[20] Dawood S, Austin L, Cristofanilli M (2014). Cancer stem cells: implications for cancer therapy. Oncology.

[21] Reya T, Morrison SJ, Clarke MF, Weissman IL (2001). Stem cells, cancer, and cancer stem cells. Nature.

[22] O’Brien CA, Kreso A, Dick JE (2009). Cancer stem cells in solid tumors:anoverview Semin. Radiat Oncol.

[23] Singh SK (2003). Identification of a cancer stem cell in human brain tumors. Cancer Res.

[24] Liu JC, Deng T, Lehal RS, Kim J, Zacksenhaus E (2007). Identification of tumorsphere- and tumor-initiating cells in HER2/Neu-induced mammary tumors. Cancer Res.

[25] Zhang S (2008). Identification and characterization of ovarian cancer-initiatingcells from primary human tumors. Cancer Res.

[26] Singh SR (2013). Cancer Lett.

[27] Lei D, Wang H, Leya H (2008). CD44 Is of functional importance for colorectal cancer stem cells. Clin Cancer Res.

[28] Takaishi S, Okumura T, Shuiping T (2009). Identification of gastric cancer stem cells using the cell surface marker CD44. Stem Cells.

[29] Vlashi E, Pajonk F (2015). Cancer stem cells, cancer cell plasticity and radiation therapy. Semin Cancer Biol.

[30] Morrison, R. et al. Targeting the mechanisms of resistance to chemotherapy and radiotherapy with the cancer stem cell hypothesis. J. Oncol. 2011,941876. 10.1155/2011/941876 PMC2958340 20981352

[31] Szakács G, Annereau J-P, Lababidi S (2004). Predicting drug sensitivity and resistance: profiling ABC transporter genes in cancer cells. Cancer Cell.

[32] Lukaszewicz AI, McMillan MK, Kahn M (2010). Small molecules and stem cells potency and lineage commitment: the new quest for the fountain of youth. J Med Chem.

[33] Kahn M (2011). Symmetric division versus asymmetric division: a tale of two coactivators. Future Med Chem.

[34] Lenz HJ, Kahn M (2014). Safely targeting cancer stem cells via selective β-catenin coactivator antagonism. Cancer Sci.

[35] Gao F, Alwhaibi A, Sabbineni H (2017). Suppression of Akt1-β-catenin pathway in advanced prostate cancer promotes TGFβ1-mediated epithelial to mesenchymal transition and metastasis. Cancer Lett.

